# A rare case of chronic stridor and dysphonia in an adult patient

**DOI:** 10.1590/S1808-86942012000200023

**Published:** 2015-10-20

**Authors:** Flávio Maria Nobre Othon Sidou, João Aragão Ximenes Filho, André Alencar Araripe Nunes, Sebastião Diógenes Pinheiro

**Affiliations:** a MD (resident). Graduated at the Federal University of Ceará (Resident at the ENT service of the Walter Cantídio University Hospital of the Federal University of Ceará); b PhD in Medicine at the Medical School of the University of São Paulo (Adjunct Professor in the Department of Surgery of the Medical School of the Federal University of Ceará); c Specialist in Head and Neck Surgery, Peroral Endoscopy, and Otorhinolaryngology (Assistant Professor in the Department of Surgery of the Medical School of the Federal University of Ceará and Head of the ENT Service of the Walter Cantídio University Hospital); d PhD in Medicine at the Medical School of the University of São Paulo (Associate Professor in the Department of Surgery of the Medical School of the Federal University of Ceará)

**Keywords:** dysphonia, dyspnea, foreign bodies, larynx

## INTRODUCTION

Chronic dysphonia and stridor are frequently seen symptoms in ENT patients. This paper describes a rare case of chronic dysphonia and stridor in an adult patient and discusses topics pertaining to differential diagnosis.

## CASE PRESENTATION

The patient is a 58-year-old male farmer, with a history of seven years of persistent dysphonia and stridor which started after he choked during a meal. He had no fever but lost 4 Kg of bodyweight. The patient was recently diagnosed with upper respiratory tract infection, which worsened his dysphonia and added dry cough, dyspnea at rest, and biphasic stridor to his symptoms. He took several medications and sought our service after being unable to attain symptom relief. The patient drinks and smokes. His socioeconomic status is precarious. Physical examination: patient is thin, shows marked biphasic stridor, diffuse hissing, and has normal signs under neck palpation. Video laryngoscopy: inter-arytenoidal edema; glottal inflammation; leukoplakia in the mid third of the right vocal fold; granulomatous site in the left vocal process; yellowish crusted lesion involving the entire sub-glottal circumference with marked lumen reduction; laryngeal mobility is preserved.

**Figure 1:**
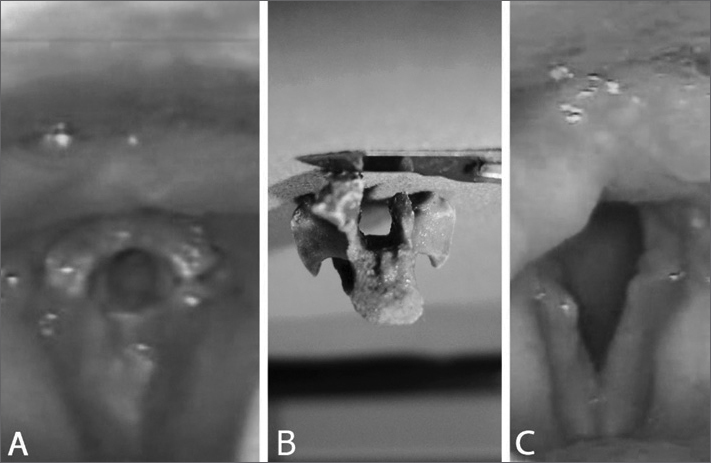
A: impacted chicken vertebra under the patient's glottis mimicking a granulomatous or neoplastic lesion. Glottal space perfectly matches the vertebra's medullary cavity. B: vertebra after removal, partially fractured during the procedure. C: video laryngoscopy showing important edema on the posterior wall and glottal inflammation.

The patient was hospitalized for an emergency tracheostomy, incisional biopsy, and imaging procedures. During suspension laryngoscopy a hard lesion was visualized and removed in block. After cleaning the site a foreign body resembling a bird's neck vertebra was seen, later confirmed by the patient as a chicken bone (Figure 1). Histopathology: bone similar to a vertebra measuring 1.7 × 1.2 cm. Histology examination: mature trabecular bone and devitalized intra-trabecular spaces.

## DISCUSSION

The main diseases with which chronic stridor and dysphonia are associated are tumor, chronic laryngitis (e.g.: laryngeal tuberculosis) and, in rare occasions, presence of a foreign body.

Laryngeal cancer is commonly seen in smokers and drinkers aged between 50 and 60. The most frequently seen manifestation is gradual chronic dysphonia (glottal tumors), dysphagia, and otalgia (supraglottal tumors). Stridor and dyspnea may occur in cases of advanced disease^1^.

Inflammatory granulomatous laryngitis, albeit rare, cannot be neglected as diagnosis is produced. Clinical presentation varies, and dysphonia, stridor, dyspnea, odynophagia, dysphagia may occur. In a third of the cases, however, the disease may be asymptomatic. In Brazil, the main etiologies are *Mycobacterium tuberculosis, Leishmania brasiliensis and Paracoccidioidis brasiliensis.* Mean time between symptom onset and diagnosis is seven months. The degree of tissue destruction, the disease's silent progress, and the little suspicion physicians usually attribute to this disease lead to significant laryngeal sequelae, among which dysphonia stands out^2^.

Laryngeal tuberculosis patients have stridor in 3.8% of the cases and dysphonia in 84.6%. The glottis is the main site of involvement in 80.8% of the cases, while the subglottis is involved in 3.8% of the patients. Video laryngoscopy findings are ulceration (50%), granuloma (50%), leukoplakia (38.5%) and non-specific inflammation (26.9%). Combined findings are described in 53.8% of the cases^3^.

Laryngeal foreign bodies present a rare, dramatic circumstance with prevalence rates ranging from 2% to 11% in cases where the airways are involved. Children are affected more frequently, especially between 6 months and 3 years of age. Diagnosis can be reached through clinical history and video laryngoscopy and confirmed by suspension laryngoscopy with the patient under general anesthesia for foreign body removal^4^. Depending on the position of the FB in the larynx and on the risk of it shifting to lower airways during intubation, a tracheostomy may be required or the FB may have to be removed with the patient under sedation and local anesthesia^5^, ^6^.

Reports of aspiration are seen in 85% of the cases. Most FBs are seen in the hypopharynx^5^ or pass through the glottis to impact against bronchi 5, 6. Bone fragments in upper airways usually produce low fever, occasional respiratory obstruction, or may be mistaken for viral infection^5^. We could not find any cases of laryngeal FB in the literature with impaction time greater than the one reported herein.

## CLOSING REMARKS

Although rare, chronically impacted upper airway foreign bodies in adults may occur and should always be considered in cases of chronic stridor and dysphonia.
